# Spatial analysis of malaria cases and *Anopheles* species in East Java region, Indonesia

**DOI:** 10.1186/s41182-024-00662-9

**Published:** 2024-12-02

**Authors:** Ngadino, Winarko, Demes Nurmayanti, Marlik, Slamet Wardoyo, Syarifah Nurhayati, Desya Wuryaningtyas

**Affiliations:** 1 Department of Environmental Health, Poltekkes Kemenkes Surabaya, Surabaya, Indonesia; 2 Disease Prevention and Control Division, East Java Health Office, Surabaya, Indonesia; 3 Bidang Pencegahan Dan Pengendalian Penyakit, Dinas Kesehatan Jawa Timur, Surabaya, Indonesia

**Keywords:** Malaria, *Anopheles*, Climate change, Epidemiology

## Abstract

Malaria remains a significant public health challenge worldwide, including in Indonesia, particularly in the East Java region. This study aimed to analyse the spatial distribution of malaria cases and *Anophele*s species that act as vectors in the area. Using an observational design with a cross-sectional approach, data on malaria cases were collected from tiers from the Community Health Centre, District Health Office and Province, all of which were documented on the Ministry of Health's malaria information system for the period 2021–2023. Malaria Vector Distribution Data from the East Java Health Office and the research team directly. Sampling of mosquitoes and larvae was carried out by researchers using a purposive sampling method, which prioritised locations with districts that have a high risk factor for the presence of breeding *Anopheles *sp., namely, Treggalek District, Malang District and Pacitan District with diverse topography, such as coastal (lagoon), rice fields and hills. The results of the analysis show that the distribution of imported malaria cases in East Java is uneven, with hotspots identified in several areas that have working population mobility from outside the East Java region. The presence of *Anopheles* species, particularly *An. sundaicus* and *An. maculatus*, contributes to their potential as vectors of malaria transmission, with *An.* *sundaicus* being more common in coastal areas and *An. maculatus* in valley and paddy fields. Environmental factors, such as topography of the region, temperature, humidity, and rainfall, influence the variation of *Anopheles *species. This study emphasises the importance of an ecosystem-based approach to malaria control, as well as the need to improve access to health services and community education. The findings provide important insights for the development of more effective and sustainable health policies in an effort to maintain malaria elimination areas in East Java.

## Introduction

Malaria remains a significant public health challenge globally, particularly in tropical regions like Southeast Asia. The World Health Organization (WHO) reported in 2021 that there were approximately 241 million malaria cases worldwide, with an estimated 627,000 deaths, predominantly in sub-Saharan Africa but also significantly affecting Southeast Asia [34]. In Indonesia, malaria continues to be a pressing issue, especially in regions like East Java, where the ecological conditions favor the proliferation of Anopheles mosquitoes, the primary vectors of the malaria parasite.

East Java is a region characterized by diverse topography, including coastal areas, lowlands, and highlands, each offering unique habitats for different *Anopheles* species [5, 12]. This ecological diversity complicates the control and eradication efforts as different species may exhibit varying behaviors, breeding sites, and susceptibility to control measures. Understanding the spatial distribution and the specific *Anopheles* species involved in malaria transmission is crucial for developing targeted intervention strategies.

Despite ongoing efforts to combat malaria, the disease persists due to several factors, including environmental changes, vector behavior, and human activities. For instance, deforestation, agricultural practices, and urbanization can alter mosquito habitats, leading to changes in the distribution and abundance of *Anopheles* species [25]. Additionally, resistance to insecticides among mosquito populations poses a significant challenge to vector control programs [18]. Therefore, there is a need for continuous monitoring and analysis of malaria cases and *Anopheles* species distribution to adapt and optimize control strategies effectively.

Recent advancements in spatial epidemiology and geographic information systems (GIS) have revolutionized the study of infectious diseases, including malaria. Spatial analysis allows researchers to visualize and analyze the distribution of disease cases and vector populations in relation to environmental and socio-economic factors [23]. These tools provide insights into the spatial patterns and hotspots of malaria transmission, which are essential for designing and implementing effective control measures.

Several studies have applied spatial analysis to understand malaria dynamics in different regions. For instance, a study in Ghana used spatial statistics to identify malaria hotspots and their relationship with environmental factors, such as temperature, rainfall, and land use [10]. Similarly, in Kenya, researchers used GIS to map the distribution of Anopheles mosquitoes and malaria cases, revealing significant spatial clustering and associations with specific ecological features [27].

In Indonesia, spatial analysis of malaria has also gained traction. A study in West Papua utilized remote sensing and GIS to map malaria incidence and associated environmental factors, highlighting the importance of vegetation and water bodies in mosquito breeding [13]. Another study in Kalimantan explored the spatial–temporal distribution of malaria and its relationship with climatic variables, providing valuable information for predicting malaria outbreaks [32].

Despite these advances, there is still a need for spatial analyses of malaria in East Java. While some studies have investigated malaria incidence in specific districts or cities within the province, comprehensive spatial analysis encompassing the entire region and integrating multiple *Anopheles* species is lacking [5, 20]. Understanding the spatial distribution of both malaria cases and the specific *Anopheles* species involved is critical for developing tailored and effective control strategies. The current body of research underscores the importance of spatial analysis in understanding malaria transmission and guiding control efforts. However, there are notable gaps, particularly in the context of East Java, Indonesia. While some localized studies provide insights into malaria incidence and vector distribution, there is a lack of comprehensive spatial analysis covering the entire province and incorporating multiple *Anopheles* species.

## Methods

### Research design

This study used an observational design with a cross-sectional approach. This method was chosen to allow data collection at one point in time, allowing analysis of the spatial distribution of malaria cases and *Anopheles* species in the East Java region [8]. This approach is in line with the research objectives to understand the spatial distribution patterns and environmental factors that influence malaria incidence in the region.

### Research location

The study was conducted in East Java province, Indonesia, which has wide topographic and environmental variations. This location was chosen due to the high incidence of imported malaria in some areas and the diversity of *Anopheles* species that act as malaria vectors [5, 31].

### Population and sample

The population of this study was all malaria cases reported in East Java during the 2021–2023 period. Data on imported malaria cases were obtained from the East Java Provincial Health Office and recorded in the Ministry of Health's malaria information system. Anopheles mosquito samples were collected from surveillance data of the East Java Provincial Health Office and the research team conducted in various locations in East Java. Sampling of mosquitoes and larvae was conducted by researchers using a purposive sampling method, which prioritised locations with districts that have high risk factors, namely, Treggalek District, Malang District and Pacitan District [16].

## Data collection

### Malaria case data

Data on malaria cases, which are all imported cases in the country, were obtained from the East Java Provincial Health Office and recorded in the Ministry of Health's malaria information system. Data collected included patient demographic information (age, gender), date of diagnosis, and location of residence [11, 12, 26].

### *Anopheles* species data

Data collection on Anopheles mosquitoes was conducted using entomological surveillance methods. The methods used included capturing adult mosquitoes, as well as sampling larvae from aquatic habitats [14]. Mosquitoes caught were then identified to species using morphological identification keys and molecular methods [7]. Existing Anopheles data were obtained from the East Java Health Office Malaria Vector Distribution Data and the direct research team. The research team captured mosquitoes and larvae in areas with diverse topography, such as coastal (lagoon), rice fields, and hills, namely, Trenggalek, Malang and Pacitan districts.

## Data analysis

Spatial analyses were conducted using Geographic Information System (GIS) software, such as ArcGIS. This analysis included mapping the distribution of malaria cases and *Anopheles* species, identifying malaria hotspots, as well as clustering analysis to determine areas with high malaria incidence.[2].

## Research ethics

This study has received approval from the Health Research Ethics Committee of the Poltekkes Kemenkes Surabaya. All patient data used in this study were kept confidential and only used for research purposes. Data collection and analysis were conducted in accordance with the principles of research ethics [17, 22, 28].

## Results

Table 1 shows the characteristics of malaria cases in East Java for three consecutive years, namely, 2021, 2022, and 2023. In terms of gender, there is a significant dominance of malaria cases among men. In 2021, out of a total of 214 cases, 200 were male (93.46%), while only 14 cases (6.54%) were female. The proportion of males remained high in 2022 with 547 out of 579 cases (94.77%), and although there was an increase in the number of female cases to 32 (5.23%), the percentage was still very small. In 2023, the number of male cases decreased slightly to 526 (90.98%), but the number of female cases increased to 52 (9.02%), indicating an increasing trend of malaria cases among women. In terms of age, the 20–45 age group is the most dominant in malaria cases. In 2021, this group recorded 186 cases (86.92%), and although the number decreased to 494 cases (85.32%) in 2022, the proportion remained high. However, in 2023, the number of cases in this age group decreased again to 430 (74.40%). Meanwhile, the 46 to 64 age group showed a significant increase, from 21 cases (9.81%) in 2021 to 103 cases (17.82%) in 2023. This indicates a shift in the age distribution of malaria cases, with older age groups experiencing an increase in proportion.

**Table 1:** Characteristics of malaria cases

	2021		2022		2023	
	n	%	n	%	n	%
Gender						
Men	200	93.46	547	94.77	526	90.98
Female	14	6.54	32	5.23	52	9.02
Age (years)						
< 10	1	0.47	2	0.35	7	1.21
10–14	1	0.47	1	0.17	3	0.52
15–19	4	1.87	15	2.59	28	4.84
20–45	186	86.92	494	85.32	430	74.40
46–64	21	9.81	65	11.22	103	17.82
> 64	1	0.47	2	0.35	7	1.21
Total	214	100	579	100	578	100

**Fig. 1:**
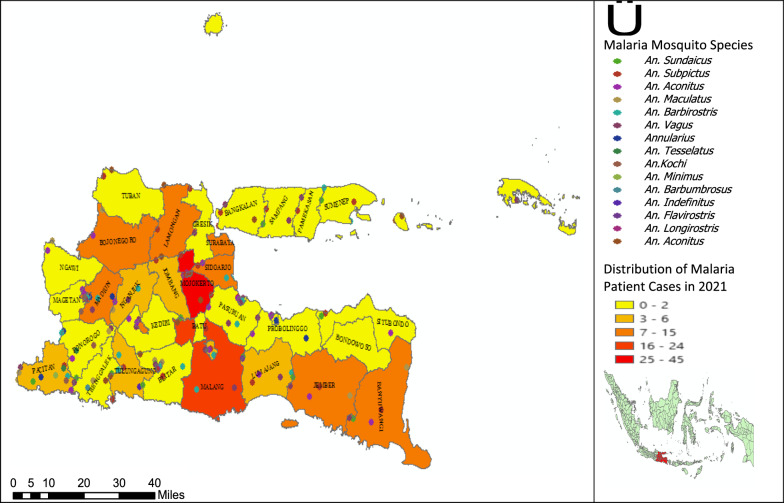
Distribution of malaria cases and presence of malaria mosquito species in East Java in 2021

Figure 1 shows that in 2021, although there was a high concentration of malaria cases in certain areas, such as Mojokerto, Madiun and Malang and followed by several other areas, namely, Surabaya and Trenggalek, these cases were imported cases from outside the East Java region, where people generally have high mobility outside the area. On the other hand, in the East Java region there are still various *Anopheles* species such as *Anopheles sundaicus* and *An. Subbpictus, An. Maculatus* and several other species in areas that have topographical areas favourable for *Anopheles *sp. breeding. Such as in Malang, Banyuwangi, Trenggalek, Pacitan and Madiun with the topography of coastal areas (lagoon), rice fields, hills and forests. The existence of *Anopheles *sp. is a concern when imported cases are found, so it has the potential to become an indiginous case.

**Fig. 2:**
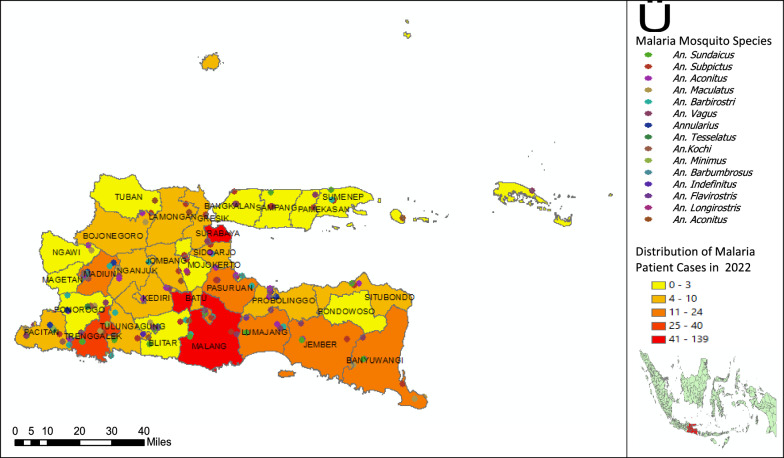
Distribution of malaria cases and the presence of malaria mosquito species in the East Java region in 2022

In 2022, the distribution pattern of malaria cases has shifted with the highest concentration of cases in Madiun, Malang and Surabaya, but all cases are still imported cases. This emphasises the need for stricter monitoring and control of population travel and migration. The presence of *Anopheles *sp. species distribution is still as in 2021 where many species variants are found in areas with coastal topography, rice fields, mountains, forests and rice fields. Malaria cases, even though they are imported cases, have the potential to become local transmission where external factors remain the main cause in the spread of malaria. This suggests that control strategies should include monitoring the movement of people and the potential importation of cases from other areas (Fig. 2).

**Fig. 3:**
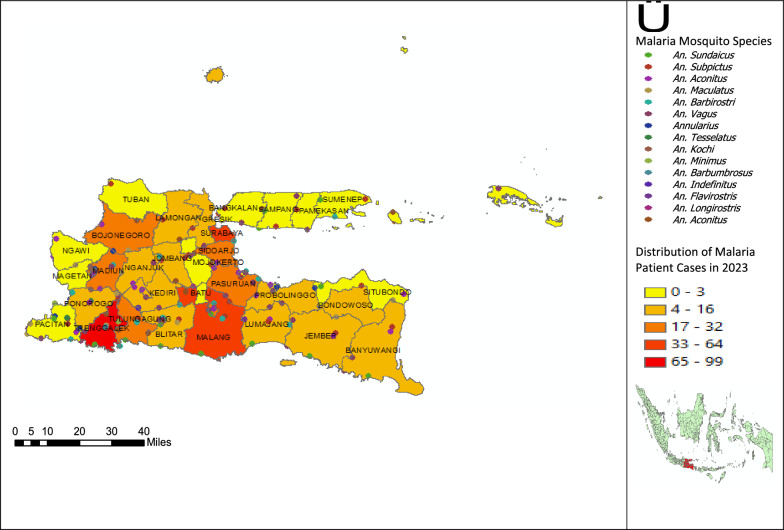
Distribution of malaria cases and the presence of malaria mosquito species in the East Java region in 2023

Figure 3 shows that although the distribution pattern of malaria cases remains consistent with previous years, with hotspots in the same areas, most of the malaria cases reported still come from outside the East Java region. This suggests that despite the presence of malaria mosquito species in East Java, malaria transmission in the region is more influenced by external factors, such as migration and travel from endemic areas. Therefore, malaria control approaches should consider these factors and involve collaboration across regions to prevent the introduction of new cases.

## Discussion

The results of this study show that the spatial distribution of imported malaria cases in East Java is uneven and tends to be concentrated in certain areas. Malaria hotspots were identified mainly in areas where the mobility of the population works from outside malaria-endemic areas. However, the discovery of *Anopheles* species was found in almost all regions of East Java. The diversity of *Anopheles* species was found in areas with favourable conditions, such as coastal areas, rice fields, and hills. This finding is a risk factor for areas with more varied *Anopheles* species to spread malaria. This finding is in line with previous studies showing that malaria distribution is often correlated with environmental factors, such as altitude, rainfall, and land use [6, 33]. The results showed that some areas still found vectors and the environment is favourable for breeding *Anopheles* sp*.,* so that these conditions have the opportunity to become indiginous transmission.

This study identified that areas that have coastal lines, rice fields, and hills tend to find varied *Anopheles* species, such as Treanggalek, Pacitan, Belitar, Lumajang, Jember. As research conducted by Hinne et al. This is supported by the research of Hinne et al., who also found that coastal areas with mangrove forests and irrigated rice fields in the region provide ideal habitat for Anopheles mosquitoes [19]. In addition, coastal areas such as the region also show the presence of vectors which can be attributed to high rainfall patterns and the presence of forests that serve as breeding grounds for mosquitoes [30]. In addition to environmental factors, socioeconomic factors such as population density and access to health services influence the distribution of malaria. Areas with high population density tend to have a higher risk of transmission due to more intense human–mosquito contact [24].

Further analysis revealed that environmental factors such as temperature, humidity, and rainfall play an important role in the spatial distribution of malaria vectors in East Java. Warm temperatures and high humidity create ideal conditions for the life cycle of Anopheles mosquitoes, while high rainfall creates stagnant water used as egg-laying sites [3]. Study by Abiodun et al. [1] suggests that increasing global temperatures and changing rainfall patterns due to climate change may expand malaria endemic areas in the tropics.

Spatial distribution analysis showed that *Anopheles sundaicus* was found more in coastal areas, such as Banyuwangi and Jember, while *Anopheles maculatus* was more dominant in inland areas, such as Bondowoso. This distribution reflects the ecological adaptation of each species to different habitats [15]. In addition, variations in the distribution of *Anopheles* species can also be affected by environmental changes due to human activities, such as deforestation and land use change [4].

The results of this study have several policy implications for malaria control in East Java, including the importance of an ecosystem-based approach to malaria control. Interventions that take into account local environmental conditions such as mosquito habitat and rainfall patterns may be more effective in reducing mosquito populations and malaria transmission [4]. Improving capacity and access to health services in areas with high malaria incidence is critical. Effective health education and outreach programmes can improve community understanding of malaria prevention and the importance of seeking immediate treatment [21, 29]. In addition, strengthening health systems to rapidly detect and respond to malaria cases can reduce the spread of the disease [35]. In addition, there is a need to develop and implement sustainable and adaptive control strategies. Given the presence of insecticide resistance, further research is needed to develop alternative control methods such as the use of new insecticides or biological approaches such as the release of sterile mosquitoes [18].

This study has several limitations that need to be considered in the interpretation of the results, i.e. the malaria case data used in the analysis may not include all cases occurring in East Java, due to limitations in reporting and access to health services. Data collection on *Anopheles* species may not cover all potential habitats, so the spatial distribution obtained may not fully represent the mosquito population present [9]. In addition, this analysis does not take into account detailed socioeconomic factors that may influence the risk of malaria transmission. Further studies that combine socioeconomic data with environmental and epidemiological data are needed to gain a more comprehensive understanding of the determinants of malaria transmission in East Java.

## Conclusion

This study provides important insights into the spatial distribution of malaria cases and *Anopheles* species in East Java. The results show that malaria distribution is influenced by various environmental and socioeconomic factors. The distribution of *Anopheles* species found showed different regional characteristics, and showed variation in the distribution of *Anopheles* species. Policy implications of this study emphasise the importance of ecosystem-based approaches, improved access to health services, and the development of sustainable control strategies. The findings also highlight the need for a multi-sectoral approach and further research to address the challenges of maintaining malaria elimination areas. With a better understanding of the spatial dynamics of malaria and mosquito vectors, control efforts can be more effective and have a positive impact in reducing risk factors for local malaria transmission in East Java.

## Data Availability

The data sets used and/or analyzed during the current study are available from the corresponding author on reasonable request.
